# Kairin

**Published:** 1884-06

**Authors:** 


### Kairin.

The *Lancet*, April 14, 1883, says that Filehne, in a recent number of the *Berliner Klinische Wochenschrift*, calls attention to the value of derivatives of chinolin which he, with Fisher and Konig, has found of great value as an anti-pyretic. These are kairin, kairolin, and finally chinolinæ hydrate of Wishnegradsky. Of these, kairin seems most likely to be of permanent value as an anti-pyretic. The muriate of kairin is a crystalline, clear, grayish-yellow powder, soluble in water, having a bitter, saltish, aromatic taste, which is disagreeable to some patients, and is, therefore, given in wafers, with a subsequent drink of water. Filehne gives five to seven grains every hour or hour and a half. The remedy has shown a marked control over the temperature of croupous pneumonia. The urine, when kairin is being given, becomes dark green.

